# Clinical and Electrophysiological Outcomes of Left Bundle Area Pacing Compared to Biventricular Pacing: An Updated Meta-analysis

**DOI:** 10.19102/icrm.2024.15053

**Published:** 2024-05-15

**Authors:** Harini Lakshman, Medhat Chowdhury, Ammar Ahmed, Everett Woods, George Flemengos, Claudine Abdou, Harshil Patel, Marcel Zughaib, Christopher Bradley

**Affiliations:** 1 Department of Cardiology, Ascension Providence, Southfield, MI, USA; 2 Department of Internal Medicine, Ascension Providence, Southfield, MI, USA; 3 Department of Pharmacy, University of Rochester, Rochester, NY, USA

**Keywords:** Cardiac resynchronization therapy, left bundle branch pacing, physiologic pacing

## Abstract

Left bundle branch area pacing (LBBAP) is a novel pacing strategy that uses the conduction system distal to the left bundle branch block level for direct activation of the left bundle and right ventricular myocardium. Our meta-analysis compared the structural, electrophysiological, clinical, and procedural outcomes of LBBAP and biventricular pacing (BVP). The meta-analysis included two randomized controlled trials and showed significant reductions in the left ventricular (LV) systolic and diastolic volumes with LBBAP compared to BVP, together with statistically significant reductions in the QRS duration, New York Heart Association (NYHA) functional class, and heart failure (HF) hospitalizations. The fluoroscopic time was also significantly shorter in the LBBAP group. However, no significant change in the LV ejection fraction was noted. Procedural complications were slightly higher in the LBBAP group, albeit not to a statistically significant degree. Our findings suggest that LBBAP may be a superior alternative to standard BVP in improving the structural, electrophysiological, and clinical components of cardiomyopathy, including the NYHA class and HF hospitalizations. LBBAP is a more physiological pacing strategy that results in normal ventricular activation and may be a viable alternative to BVP for cardiac synchronization therapy.

## Introduction

Cardiac resynchronization therapy (CRT) in patients with left ventricular (LV) systolic dysfunction and concomitant left bundle branch block (LBBB) has been shown to lower the risk of hospitalization for heart failure (HF) and all-cause mortality.^1^ Current guidelines recommend the use of biventricular pacing (BVP) for the correction of electromechanical dyssynchrony in patients with reduced ejection fraction.^1^ However, 30% of patients are non-responders to BVP, which correlates to reduced QRS narrowing compared to the responder cohort. Additionally, BVP is noted to unify two wavefronts, leaving residual asynchrony.^2,3^

Hence, alternative modalities to correct electromechanical dyssynchrony, such as the His–Purkinje pacing and LBB pacing (LBBP), have evolved as potential alternatives to BVP.

LBBP was first described by Huang et al. in 2017. These authors demonstrated that pacing the proximal left bundle and its branches with LV septal myocardial capture is beneficial in HF patients with complete LBBB.^4^

Prior meta-analyses of observational studies have indicated favorable QRS reduction and concomitant improvement in New York Heart Association (NYHA) class symptoms in patients with LBBP compared to traditional BVP.^5^ Despite favorable results, the studies included were subject to bias considering their observational nature and geographic homogeneity (all studies were from China). The Left Bundle Branch Pacing Versus Biventricular Pacing for Cardiac Resynchronization Therapy (LBBP-RESYNC) study is the first randomized trial to compare structural, electrophysiological, and clinical outcomes in patients randomized to BVP and LBB area pacing (LBBAP). We therefore performed a meta-analysis including randomized and propensity-matched studies to evaluate the procedural and clinical outcomes of LBBP versus BVP in patients with LV systolic dysfunction and LBBB.

## Methods

As a meta-analysis, this research did not require ethics committee approval. Throughout the stages of conception, study design, data analysis, and results, this meta-analysis was prepared in concordance with the Preferred Reporting Items for Systematic Reviews and Meta-analyses and the Cochrane Handbook guidelines. No patients or members of the public were involved in the design, conduct, reporting, and dissemination plans of our research.

### Literature search and selection criteria

We conducted an online literature search in PubMed, Cochrane, and Google Scholar from October 2022 to identify relevant studies. The search terms were “left bundle branch pacing,” “biventricular,” “left bundle branch area,” and “cardiac resynchronization therapy,” which were used alone and in combination. In addition to looking through eligible studies in the reference lists of the retrieved papers and relevant reviews, studies were included if they involved patients who underwent LBBP and were compared to cases of BVP. We excluded any systematic reviews or publications with overlapping data sources and outcomes not comparable to the outcomes tested in our meta-analysis. All studies were required to be available in the English language.

### Data extraction and quality assessment

The following information was extracted from the selected studies: first author, publication year, number of patients enrolled, age, sex, NYHA class, presence of ischemic cardiomyopathy, LV end-diastolic and end-systolic volumes (LVEDV, LVESV), ejection fractions, baseline QRS time, lead placements, and medication use (including β-blockers, diuretics, angiotensin-converting enzyme inhibitors/angiotensin receptor blockers, and aldosterone antagonists).

### Statistical analysis

Categorical variables are reported as counts and percentages, and continuous variables are reported using mean ± standard deviation values. For continuous variables, differences in two groups were assessed using the *t* test. For categorical variables, Fisher’s exact test was used. Statistical analyses were performed using RevMan version 5.3 (Cochrane, London, UK).

## Results

### Study identification

**Figure 1:**
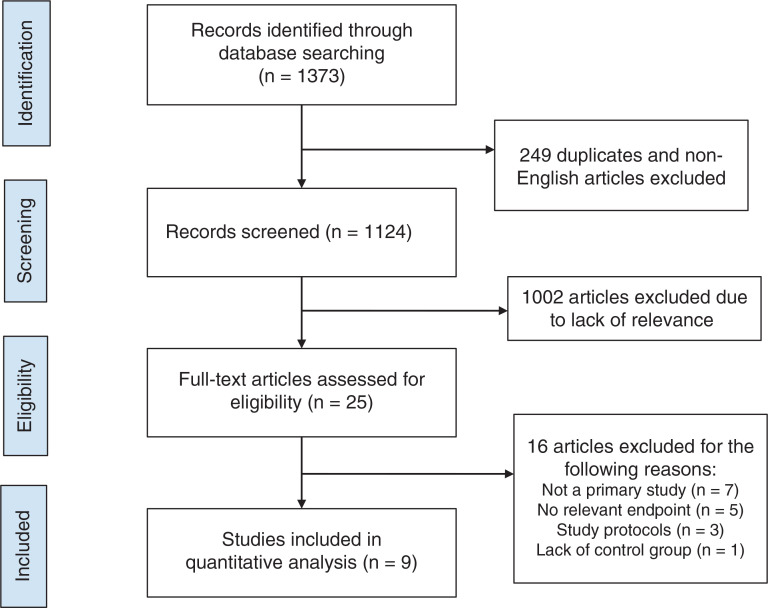
Study selection process.

We identified a total of 1372 studies using the search methodology described earlier. After excluding duplicates, case reports, review articles, observational trials without propensity matching, and irrelevant search results, 24 articles were included. After a thorough review of studies and based on desired outcomes, two randomized controlled trials and seven observational studies, which were propensity matched, were included for the final analysis analysis **(Figure 1)**.

### Study characteristics

A total of nine studies were included in this meta-analysis, of which two were randomized controlled trials^6,7^ and the other seven were prospective non-randomized controlled studies propensity matched for age, sex, and history of ischemic heart disease; NYHA class for shortness of breath; QRS duration, LV end-diastolic dimension, and LV ejection fraction (LVEF) at baseline; and medications for HF.^4,8–10^ A total of 746 patients were included, with 392 in the LBBP arm and 354 in the BVP arm. The characteristics of the individual studies are listed in **Table 1**.

**Table 1:** Baseline Characteristics of Included Studies

Author	Type of Study	Year	Number of Patients		Mean Age (Years)		Male sex (%)		LVEF (%)		QRSd (mm)		Follow-up (Months)
			LBBAP	BVP	LBBAP	BVP	LBBAP	BVP	LBBAP	BVP	LBBAP	BVP	
Wang et al.^6^		2022	20	20	62.3	65.3	35	65	28.3	31.1	174.6	174.7	6
Pujol-Lopez et al.^7^	Randomized controlled trial	2022	35	35	65.7	68.1	65.7	72.4	27	28	177	178	6
Vijayaraman et al.^8^		2022	114	44	69	65	59.6	82	29.7	26.5	166.7	181.9	30
Li et al.^9^		2020	27	54	57.5	58.5	51.9	61.1	28.8	27.2	178.2	180.9	6
Chen et al.^21^		2019	49	51	67.14	64.37	24	30	29.05	28.36	180.12	175.7	12
Wang et al.^22^		2020	10	30	64.8	62.9	90	76.7	26.8	26.4	183.6	174.6	6
Vijayaraman et al.^23^		2023	981	797	68	69	63.1	69	26	26	160	160	33
Guo et al.^24^		2020	21	21	66.1		42.9	42.9	30	29.8	167.7	163.6	6
Wu et al.^25^	Randomized controlled trial	2020	32	54	67.2	68.3	43.8	53.7	30.9	30	166.2	161.1	12
Total numbers			1289	1106									

*Abbreviations:* BVP, biventricular pacing; LBBAP, left bundle branch area pacing; LVEF, left ventricular ejection fraction; QRSd, QRS duration.

### Outcomes

#### Structural

**Figure 2:**
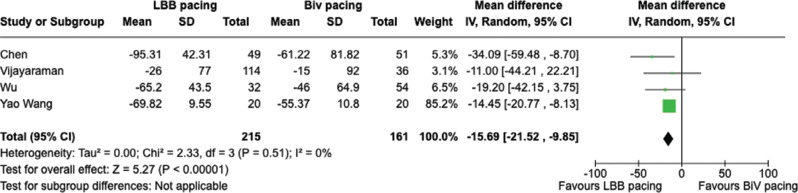
Left ventricular end-diastolic volume change. *Abbreviations*: BVP, biventricular pacing; CI, confidence interval; IV, weighted mean difference; LBBAP, left bundle branch area pacing; SD, standard deviation.

**Figure 3:**
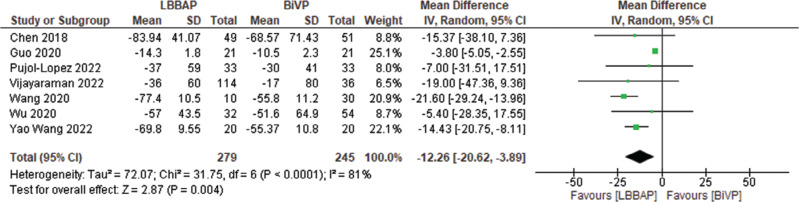
Left ventricular end-systolic volume change. *Abbreviations:* BVP, biventricular pacing; CI, confidence interval; IV, weighted mean difference; LBBAP, left bundle branch area pacing; SD, standard deviation.

**Figure 4:**
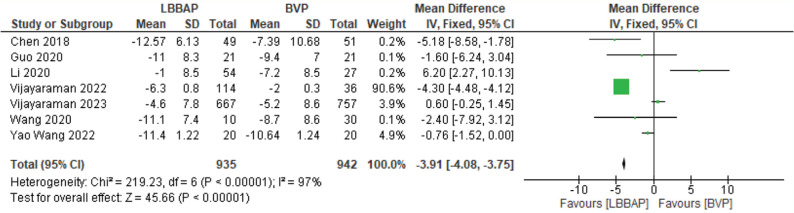
Left ventricular end-diastolic diameter change. *Abbreviations:* BVP, biventricular pacing; CI, confidence interval; IV, weighted mean difference; LBBAP, left bundle branch area pacing; SD, standard deviation.

Structural outcomes such as left ventricular end-diastolic diameter (LVEDD), LVESV, and LVEDV were studied across trials reporting relevant results. There were significant changes in LVEDV and LVESV with mean decreases of 15.69 and 12.26 mm^3^, respectively **(Figures 2 and 3)**. LVEDD had a significant decrease as well, with a mean of 3.9 mm **(Figure 4)**.

#### Physiological and electrophysiological

**Figure 5:**
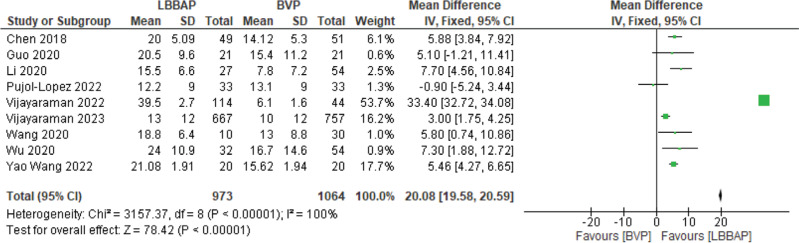
Left ventricular ejection fraction change. *Abbreviations:* BVP, biventricular pacing; CI, confidence interval; IV, weighted mean difference; LBBAP, left bundle branch area pacing; SD, standard deviation.

**Figure 6:**
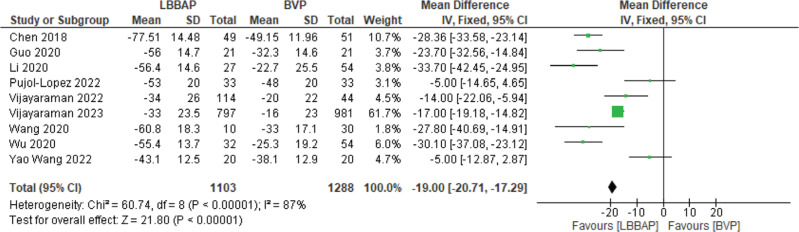
QRS duration change. *Abbreviations:* BVP, biventricular pacing; CI, confidence interval; IV, weighted mean difference; LBBAP, left bundle branch area pacing; SD, standard deviation.

The change in LVEF was measured. There was a significant improvement in LVEF by a mean of 20.08% with LBBAP **(Figure 5)**. A significant change in mean QRS of 19 ms (*P* < .00001) was also noted **(Figure 6)**.

#### Clinical

**Figure 7:**
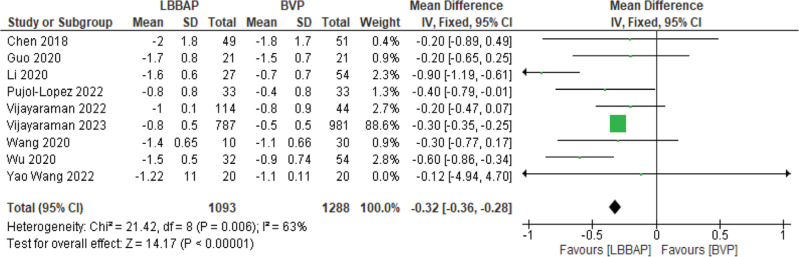
New York Heart Association functional class change. *Abbreviations:* BVP, biventricular pacing; CI, confidence interval; IV, weighted mean difference; LBBAP, left bundle branch area pacing; SD, standard deviation.

**Figure 8:**
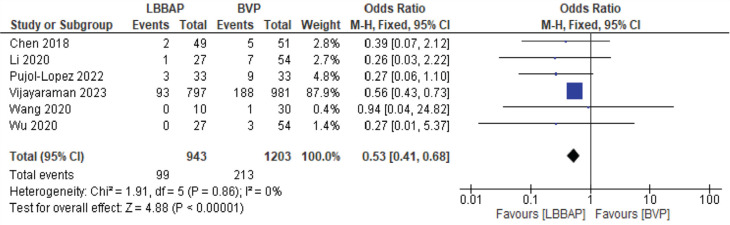
Heart failure hospitalizations. *Abbreviations:* BVP, biventricular pacing; CI, confidence interval; LBBAP, left bundle branch area pacing; M-H, Mantel–Haenszel; SD, standard deviation.

A significant change in NYHA class was noted, with a mean decrease of 0.32 **(Figure 7)**. There was also a significant decrease in the number of hospitalizations for HF, with an odds ratio of 0.53 **(Figure 8)**.

#### Procedural

**Figure 9:**
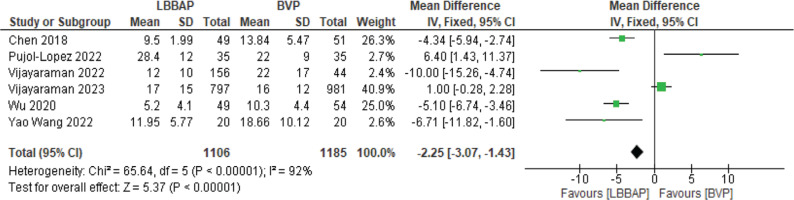
Fluoroscopic time. *Abbreviations:* BVP, biventricular pacing; CI, confidence interval; IV, weighted mean difference; LBBAP, left bundle branch area pacing; SD, standard deviation.

**Figure 10:**
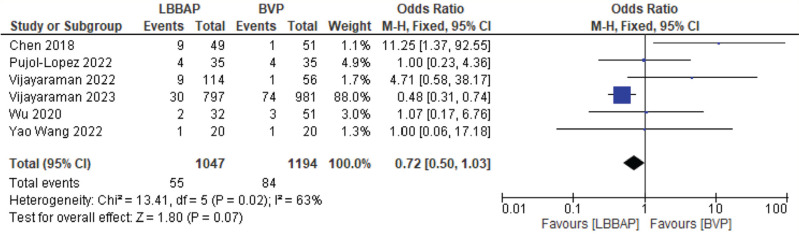
Procedural complications. *Abbreviations:* BVP, biventricular pacing; CI, confidence interval; LBBAP, left bundle branch area pacing; M-H, Mantel–Haenszel; SD, standard deviation.

The fluoroscopic time was decreased to a mean of 2.25 min with statistical significance **(Figure 9)**. There was no statistically significant difference in procedural complications; however, the trend was lower complications with LBBAP **(Figure 10)**.

## Discussion

Our meta-analysis aimed to analyze the structural, electrophysiological, clinical, and procedural aspects of LBBAP compared to BVP. A previous meta-analysis comparing LBBAP and BVP had included only observational and prospective trials. This is the first meta-analysis to include two randomized controlled trials.^6,7^

This meta-analysis reported significant reductions in LV systolic and diastolic volumes with LBBAP compared to BVP. There was a significant increase in LVEF in the LBBAP group and also a statistically significant decrease in the QRS duration, NYHA class, and HF hospitalizations. The fluoroscopic time was found to be shorter in the LBBAP group. Procedural complications, although not statistically significant, were slightly greater in the BVP group.

Given these findings, conduction system pacing may be a superior alternative to standard BVP not only in the improvement of structural and electrophysiological components but also in terms of clinical components of cardiomyopathy, including LVEF, NYHA class, and HF hospitalizations.

CRT has proven to be beneficial for patients with moderate or severe symptoms of HF, a reduced LVEF, or a prolonged QRS duration and those in sinus rhythm. The Comparison of Medical Therapy, Pacing, and Defibrillation in Heart Failure (COMPANION) and Cardiac Resynchronization—Heart Failure (CARE-HF) trials compared CRT pacemakers to placebo, and efficacy was established.^11,12^ BVP for cardiac synchronization therapy, although widely efficacious, is non-physiological. The activation involves the ventricular myocardium and not the conduction system. Hence, the QRS remains wide.^13^ There are significant rates of non-responsiveness associated with BVP, which may vary between 30% and 45% depending on the endpoint. Factors such as ischemic etiology, advanced NYHA class, severe mitral regurgitation, severe dilatation of the left atrium, and a short interventricular mechanical delay have been associated with both clinical and echocardiographic non-responsiveness.^14,15^ There are several other disadvantages, including increased periprocedural complications with coronary sinus lead placement and increased procedural and fluoroscopic times.^14^

Attempts have been made to increase physiological pacing, which includes His-bundle pacing and, more recently, LBBAP. His-bundle pacing has been extensively compared with BVP in multiple studies.^15–20^ Although selective His-bundle pacing results in normal ventricular activation, non-selective His-bundle pacing is observed with fusion from direct stimulation from the septal myocardium.^16^ With complications such as damage to the His bundle and unstable pacing threshold, the His Bundle Pacing versus Coronary Sinus Pacing for Cardiac Resynchronization Therapy (HIS-SYNC) study showed that, in a subset of patients with LBBB, His-bundle pacing was not effective.^17,18^

Direct LBBP was first reported by Huang et al. with a low pacing threshold of 0.5 V.^4^ LBBP uses the conduction system distal to the level of LBBB for direct activation of the left bundle and the right ventricular myocardium. This significantly decreases the LV peak activation time.^4^ Chen et al. demonstrated the ideal placement of leads for LBBAP to be perpendicular to the ventricular septum at a location approximately 15–20 mm below the tricuspid valve annulus under fluoroscopic imaging, primarily using 30° right anterior oblique and/or 30° left anterior oblique projections.^10,21^ Advancing the lead to the interventricular septum should result in a right bundle branch (RBB) pattern. The LV activation time is measured from the pacing stimulus to the peak of the R-wave in V5/V6, and the ideal time should be ≤80 ms. Both LBB and RBB could be corrected by left bundle pacing, resulting in a significant reduction in the QRS duration.^21^

Multiple observational trials and meta-analyses of these trials have described the efficacy of LBBP.^1,4,6,7,9,16–18^ LBBP was deemed to be clinically and electrophysiologically non-inferior if not superior to conventional pacing based on observational trials given a more physiological pacing result. A major limitation to the implant procedure is experience, as early studies were done mostly at Chinese centers, although international exposure is growing. There is a need for randomized controlled trials for further comparison. Recently, there have been randomized controlled trials performed to compare LBBP and BVP, which demonstrated electromechanical benefits, including QRS duration shortening and structural changes.^6,7^ While these studies do show significant reductions in LV volumes and QRS duration, the clinical relevance is yet to be established. Our meta-analysis does show clinical benefits with improved LVEF, NYHA class, and HF hospitalization in the LBBAP group compared to the BVP group when both randomized controlled trials and propensity-matched observational trials were included.

Our meta-analysis does have limitations with increased heterogeneity. A number of biases could be present due to the nature of the study given the inclusion of observational and randomized controlled trials. More randomized controlled trials with larger sample sizes are necessary to reliably establish efficacy and safety.

## Conclusions

When compared to BVP, LBBAP demonstrates favorable outcomes in QRS duration and structural changes that also correlate with better clinical outcomes, such as NYHA functional class and reductions in HF hospitalizations. A trend toward increased complications, although statistically insignificant, was associated with LBBP and can be explained due to the relatively new status of the procedure and possible learning curves. Further randomized controlled trials in large-volume centers are warranted to reliably establish safety.
